# Efficacy and safety of camrelizumab combined with oxaliplatin and S‐1 as neoadjuvant treatment in locally advanced gastric or gastroesophageal junction cancer: A phase II, single‐arm study

**DOI:** 10.1002/cam4.7006

**Published:** 2024-02-24

**Authors:** Wen‐Jin Zhong, Jian‐An Lin, Chu‐Ying Wu, Jiantian Wang, Jun‐Xing Chen, Huida Zheng, Kai Ye

**Affiliations:** ^1^ Department of Gastrointestinal Surgery The Second Affiliated Hospital of Fujian Medical University QuanZhou City Fujian China

**Keywords:** Camrelizumab, gastric cancer, gastroesophageal junction cancer, immune checkpoint inhibitor, Neoadjuvant therapy, S‐1

## Abstract

**Purpose:**

In the present study, we aimed to evaluate the efficacy and safety of camrelizumab combined with oxaliplatin plus S‐1 in patients with resectable gastric or gastroesophageal junction cancer.

**Methods:**

In this single‐arm, phase II clinical trial, patients with locally advanced gastric or gastroesophageal junction adenocarcinoma were enrolled to receive three cycles of neoadjuvant camrelizumab and oxaliplatin plus S‐1 every 3 weeks, followed by surgical resection and adjuvant therapy with the same regimen. The primary endpoint was pathological complete response (pCR) (ypT0) rate and secondary endpoints were R0 resection rate, total pCR (tpCR, ypT0N0) rate, major pathological response (MPR) rate, downstaging, objective response rate (ORR), disease control rate (DCR), event‐free survival (EFS), overall survival (OS), and safety.

**Results:**

Between September, 2020 and January, 2022, a total of 29 patients were enrolled in the present study, all of whom completed neoadjuvant therapy and underwent surgery. Three (10.3%) (95% CI: 2.2–27.4) patients achieved pCR as well as tpCR, 20 (69.0%) patients had MPR and 28 (96.6%) patients achieved R0 resection. Treatment‐emergent adverse events (AEs) of any grade were observed in 24 (82.8%) patients. Immune‐related adverse events of any grade were reported in 13 (44.8%) patients, whereas no grade 3 or higher adverse events occurred.

**Conclusion:**

The neoadjuvant therapy with camrelizumab in combination with oxaliplatin and S‐1 showed a modest pCR rate, and favorable MPR rate and safety profile in patients with gastric or gastroesophageal junction cancer.

## 1 INTRODUCTION

Worldwide, gastric cancer (GC) is one of the most commonly diagnosed malignancies and the fourth leading cause of cancer‐related death. In Asian countries, the incidence and mortality rates are even higher.^1^ For patients with locally advanced GC (LAGC), surgical resection is the mainstay of treatment, and it has been demonstrated that compared with surgery alone, surgery with neoadjuvant chemotherapy showed improved survival outcomes.^2^, ^3^ However, in terms of long‐term efficacy, approximately one‐third of the LAGC patients will experience recurrence within 3 years after surgical resection.^4^, ^5^ Moreover, the optimal treatment regimen has not been decided yet. Thus, exploring an effective neoadjuvant regimen to prolonged survival outcomes for LAGC and gastroesophageal junction cancer (GEJC) has become an urgent need.

In recent years, with the emergence of immunotherapy, clinical benefits have been shown when using immune checkpoint inhibitors (ICIs) monotherapy in patients with heavily pretreated unresectable advanced gastric cancer or GEJC.^6^ Moreover, the addition of ICIs to chemotherapy demonstrated improved survival outcomes compared with chemotherapy alone as first‐line treatment for patients with unresectable gastric cancer or GEJC in phase III trials.^7^, ^8^ However, the efficacy of combined regimen with ICIs and chemotherapy has not been fully explored in the neoadjuvant setting.

Camrelizumab, a programmed cell death 1 (PD‐1) inhibitor, has been approved for the treatment of a variety of solid tumors in China.^9^, ^10^, ^11^ The S‐1 plus oxaliplatin (SOX) regimen is one of the most recommended first‐line therapies for unresectable GC in Asian countries.^12^ Furthermore, the RESOLVE study showed meaningful clinical benefits in perioperative‐SOX group when treating LAGC or GEJC in Chinese population.^13^ On the basis of the above‐mentioned information, camrelizumab plus SOX regimen might be a potential neoadjuvant therapeutic option for patients with LAGC.

Therefore, in this prospective, single‐arm, phase II study, we investigated the efficacy and safety profile of combination treatment of SOX regimen and camrelizumab for patients with LAGC and GEJC.

## 2 MATERIALS AND METHODS

### 2.1 Study design and participants

This single‐arm, open‐label, phase II study (NCT05602935) was conducted at The Second Affiliated Hospital of Fujian Medical University between September, 2020 and January, 2022. The study was performed in accordance with the Guidelines for Good Clinical Practice and the Declaration of Helsinki. The study protocol was approved by the institutional ethics committees of The Second Affiliated Hospital of Fujian Medical University. Written informed consents of all patients were obtained before study enrollment.

Patients who met the following criteria were included: 1. Age older than 18 years of age; 2. With gastric or gastroesophageal junction adenocarcinoma confirmed by histological examination; 3. No history of systemic therapy; 4. Eastern Cooperative Oncology Group (ECOG) performance status 0 or 1; 5. With measurable lesions based on Response Evaluation Criteria in Solid Tumors (RECIST) version 1.1; 6. With clinical stage cT3‐4bN1‐3 M0 evaluated by computed tomography (CT) per American Joint Committee on Cancer (AJCC) staging manual 8th edition^14^; 7. Life expectancy longer than 12 months; 8. Adequate hematological, cardiac, hepatic and renal function. Exclusive criteria were patients: 1. With immunodeficiency or autoimmune disease; 2. With unresectable disease assessed by investigators; 3. With uncontrollable cardiovascular disease; 4. With other malignancies; 5. With a past medical history of interstitial lung disease or non‐infectious pneumonia; 6. With drug allergy; 7. Under pregnancy and lactation.

### 2.2 Treatment procedure

Eligible patients received camrelizumab (200 mg, iv, on day one) and oxaliplatin (130 mg/m^2^, ivgtt, on day one) plus S‐1 (40 mg [body surface area (BSA) <1.25 m^2^], 50 mg (1.25 ≤ BSA <1.5 m^2^), or 60 mg (BSA ≥1.5 m^2^), twice daily, on day 1–14) in a 3‐week cycle for three cycles as neoadjuvant treatment. Three cycles of adjuvant therapy with the same regimen in the neoadjuvant setting were administrated 4–6 weeks after surgery.

Dose reduction and re‐escalation were not allowed for camrelizumab; however, dose interruption of up to 12 weeks was permitted when patients experienced serious toxicities, after which camrelizumab was discontinued, unless investigators believed that patients could still benefit from it. Dose reduction was permitted for oxaliplatin and S‐1 when ≥3 grade hematological toxicity and/or ≥3 grade non‐hematological toxicities occurred. In addition, treatments were terminated when completion of three cycles adjuvant treatment or until disease progression or recurrence or intolerable toxicity.

After completion of three cycles of neoadjuvant treatment, eligible patients were assessed by clinicians and radiologists for surgery according to clinical practice. Surgery procedures included D2 total gastrectomy or D2 sub‐gastrectomy with or without laparoscopic or open total gastrectomy.

### 2.3 Assessment

Routine blood examination, hepatic and renal functions were monitored each cycle during the neoadjuvant phase. Biopsy samples were obtained during surgical resection and assessed for tumor downstaging based on the AJCC 8th edition. HER2 status was assessed by IHC or fluorescence in situ hybridization (FISH), and defined as positive when scored 3+ on IHC or 2+ on IHC and positive on FISH. Computed tomography (CT) and electronic gastroscope were performed before study treatment and after the completion of neoadjuvant therapy. Imaging examination was monitored every 3 months in the first 2 years, then every 6 months in the first 5 years, then once a year. The PD‐L1 expression level was evaluated using ZR‐3 antibody and graded as positive if tumor proportion score (TPS) ≥1%. AJCC 8th edition of Tumor Regression Grade (TRG) was defined as TRG0 (absence of tumor cells in both primary tumor and lymph nodes); TRG1 (single cells or rare small groups of cancer cells); TRG2 (evident cancer cells reduction but more than rare small groups); and TRG3 (no evidence of tumor regression). Safety profiles from neoadjuvant therapy initiation to 30 days after last adjuvant treatment were determined by Common Terminology Criteria for Adverse Events (CTCAE) 5.0. Meanwhile, surgical and post‐operative complications were assessed per Clavien‐Dindo classification.^15^

### 2.4 Outcomes

The primary endpoint was pathological complete response (pCR) (ypT0) rate, defined as the proportion of patients with no residual tumor cells at the primary tumor. Secondary endpoints included R0 resection rate (defined as the absence of tumor cells in resection margin), total pCR rate (tpCR, ypT0N0) (defined as the absence of tumor cells at both primary tumor and lymph nodes), major pathological response rate (MPR) (including AJCC TRG 0 and 1), downstaging defined as response of the primary tumor and lymph node metastases to previous neoadjuvant therapy, objective response rate (ORR) (including partial response [PR] and complete response [CR]), disease control rate (DCR) (including PR, CR and stable disease [SD]), event‐free survival (EFS), overall survival (OS), and safety.

### 2.5 Statistical analysis

The pCR rate is approximately 5.6% according to RESOLVE study.^13^ Exact method was used to compute confidence interval (CI). We hypothesized the pCR rate could achieve 17% for SOX plus cameralizumab. A sample size of 29 is required to produce a two‐sided 95% confidence interval of (5.7%, 35.5%), whose lower boundary is beyond 5.6%. Continuous variations were described in medians and ranges and categorical variables were described in frequencies and percentages. The efficacy and safety were evaluated in the full analysis set. The pCR, MPR, ORR, DCR, and R0 resection rate were calculated, and the corresponding CIs were estimated by Clopper–Pearson method. Statistical analyses were carried out using SAS Version 9.4.

## 3 RESULTS

### 3.1 Baseline characteristics and treatment

**FIGURE 1:**
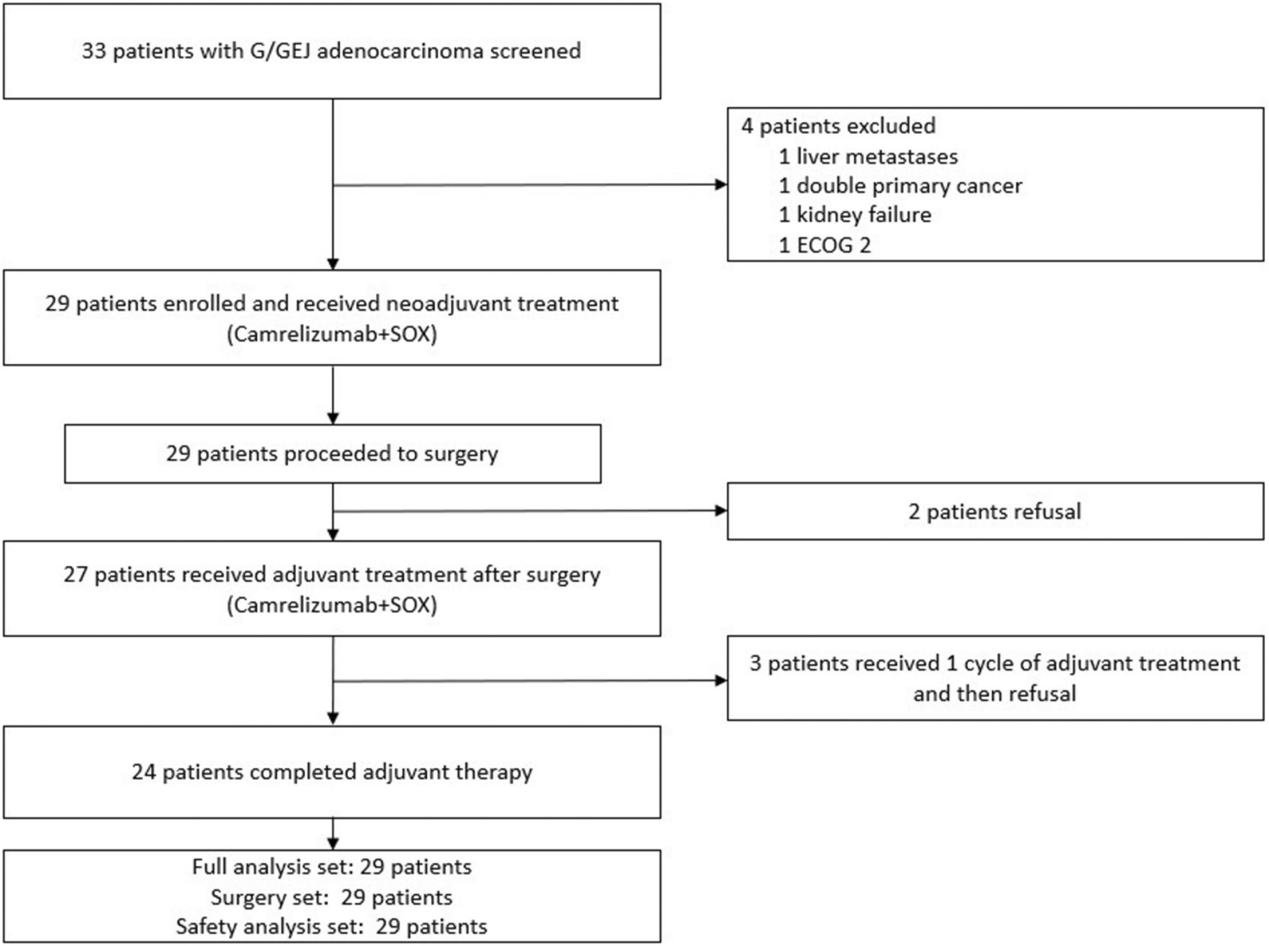
Patient flowchart. The diagram shows the progress of this study.

Between September, 2020 and January, 2022, a total of 33 patients were screened, after which 29 patients were enrolled in the present study to receive neoadjuvant therapy (Figure 1). The median age was 62 years (range 39–84), and ECOG status was 0 (19/29, 65.5%) or 1 (10/29, 34.5%). Twelve (41.4%) patients had GC, while the remaining (58.6%) patients had GEJC. Seven (24.1%) patients had signet‐ring cell carcinoma. Regarding the clinical stage, a majority (27/29, 93.1%) of patients were in stage III, whereas 2 (6.9%) patients were in stage IVA. Proficient mismatch repair (pMMR) was observed in 28 patients (96.6%), while only one patient was deficient mismatch repair (dMMR). Five patients (17.2%) were PD‐L1 positive, whereas 24 (82.8%) were PD‐L1 negative. The baseline characteristics were summarized in Table 1. All enrolled patients completed three cycles of neoadjuvant therapy and underwent surgical resection. However, two patients refused to receive adjuvant therapy due to personal reasons, three patients only received one cycle of adjuvant therapy (Figure 1).

**TABLE 1:** Baseline characteristics.

Characteristics	Patients (*n* = 29)
Age (years), median (range)	62 (39–84)
Sex, *n* (%)	
Male	18 (62.1)
Female	11 (37.9)
ECOG performance status, *n* (%)	
0	19 (65.5)
1	10 (34.5)
Primary tumor location, *n* (%)	
Gastric	12 (41.4)
GEJ	17 (58.6)
Histological type, *n* (%)	
Adenocarcinoma	29 (100)
Signet‐ring cell carcinoma	7 (24.1)
Lauren's classification, *n* (%)	
Intestinal	11 (37.9)
Diffuse	12 (41.4)
Mixed	6 (20.7)
T stage, *n* (%)	
T3	12 (41.4)
T4a	15 (51.7)
T4b	2 (6.9)
N stage, *n* (%)	
N+	29 (100)
Clinical stage, *n* (%)	
III	27 (93.1)
IVA	2 (6.9)
HER2 status, *n* (%)	
Positive	3 (10.3)
Negative	25 (86.2)
Missing	1 (3.4)
Mismatch repair status, *n* (%)	
dMMR	1 (3.4)
pMMR	28 (96.6)
PD‐L1 status, *n* (%)	
TPS≥1%	5 (17.2)
TPS<1%	24 (82.8)

Abbreviations: ECOG, Eastern Cooperative Oncology Group; GEJ, gastroesophageal junction; dMMR, deficient proficient mismatch repair; PD‐L1, programmed cell death receptor ligand 1; pMMR, proficient mismatch repair; TPS, tumor proportion score.

### 3.2 Efficacy

Overall, 29 patients who received neoadjuvant treatment underwent surgical resection, among which 23 (79.3%) patients received total gastrectomy, and the remaining 6 (20.7%) patients received subtotal gastrectomy. Finally, R0 resection was achieved in 28 (96.6%) patients. The median number of lymph nodes removed was 33 (range, 17–130). The median operative time was 150.0 min (range, 110.0–175.0). The median hospital stay was 9 days (range, 7–20) (Table 2).

**TABLE 2:** Pathological response and surgery results.

	Surgery set (*n* = 29)
AJCC‐TRG, *n* (%)	
0	3 (10.3)
1	17 (58.6)
2	5 (17.2)
3	4 (13.8)
pCR rate, *n* (%) [95% CI]	3 (10.3) [2.2–27.4]
MPR rate, *n* (%) [95% CI]	20 (69.0) [49.2–84.7]
Patients with surgery	
R0 resection rate, *n* (%)	28 (96.6)
R1 resection rate, *n* (%)	1 (3.4)
Gastrectomy, *n* (%)	
Total gastrectomy	23 (79.3)
Subtotal gastrectomy	6 (20.7)
Lymphadenectomy	
D2 dissection	29 (100.0)
Number of lymph nodes removed (*n*), median (range)	33 (17–130)
Operative time (min), median (range)	150.0 (110.0–175.0)
Postoperative anus exhausting time (hour), median(range)	35 (20–72)
Postoperative landing time (hour), median (range)	25 (12–72)
Reoperation, *n* (%)	0 (0)
Length of hospital stay (day), median (range)	9 (7–20)

Abbreviations: CI, confidence interval; MPR, major pathological response; pCR, pathological complete response; TRG, tumor regression grade; UTA, unable to access.

In terms of pathological response, 3 (10.3%) (95% CI: 2.2–27.4) patients had pCR as well as tpCR, meanwhile, 20 (69.0%) patients had MPR (Table 2). According to AJCC 8th edition, ypT0‐1 was achieved in 58.6% (17/29) patients, and ypN0 was achieved in 69% (20/29) patients. Collectively, 25 (86.2%) patients achieved overall TNM downstaging (Table 3). In total, the objective response rate (ORR) and disease control rate (DCR) were observed in 27 (93.1%) (95% CI: 77.2–99.2) patients and 29 (100%) (95% CI: 88.1–100.0) patients, respectively.

**TABLE 3:** Downstaging.

	Pre‐neoadjuvant therapy (CT) (*n* = 29)	Post‐neoadjuvant therapy (surgical pathology) (*n* = 29)
T stage, *n* (%)		
T0	0 (0)	3 (10.3)
T1	0 (0)	14 (48.2)
T2	0 (0)	8 (27.6)
T3	12 (41.4)	4 (13.8)
T4a	15 (51.7)	0 (0)
T4b	2 (6.9)	0 (0)
N stage, *n* (%)		
N0	0 (0)	20 (69.0)
N1	7 (24.1)	4 (13.8)
N2	10 (34.5)	2 (6.9)
N3	12 (41.4)	3 (10.3)
ypTNM stage, *n* (%)		
I		19 (65.5)
II		3 (10.3)
III		4 (13.8)
Change in overall stage, *n* (%)		
Downstaged		25 (86.2)
Upstaged		0 (0)
No change		4 (13.8)

In addition, subgroup analysis showed that three patients achieved pCR. Among them, two patients had primary tumors located in the gastroesophageal junction, one located in the stomach. Two patients had signet cell carcinoma. Two patients were intestinal type (Lauren's classification), one patient was diffuse type (Lauren's classification). All patients had negative PD‐L1 expression (TPS <1%) (Table 4).

**TABLE 4:** Comparison of pCR rate among subgroups.

	pCR	Non‐pCR
Primary tumor site, *n* (%)		
Gastroesophageal junction (*n* = 17)	2 (11.8)	15 (88.2)
Stomach (*n* = 12)	1 (8.3)	11 (91.7)
Signet cell carcinoma, *n* (%)		
Yes (*n* = 7)	2 (28.6)	5 (71.4)
No (*n* = 22)	1 (4.5)	21 (95.5)
Lauren's classification, *n* (%)		
Intestinal type (*n* = 11)	2 (18.2)	9 (81.8)
Diffuse type (*n* = 12)	1 (8.3)	11 (91.7)
Mixed type (*n* = 6)	0 (0)	6 (100)
PD‐L1 status, *n* (%)		
TPS <1% (*n* = 24)	3 (12.5)	21 (87.5)
TPS≥1% (*n* = 5)	0 (0)	5 (100)

Abbreviations: pCR, pathological complete response; PD‐L1, programmed cell death receptor ligand 1; TPS, tumor proportion score.

**FIGURE 2:**
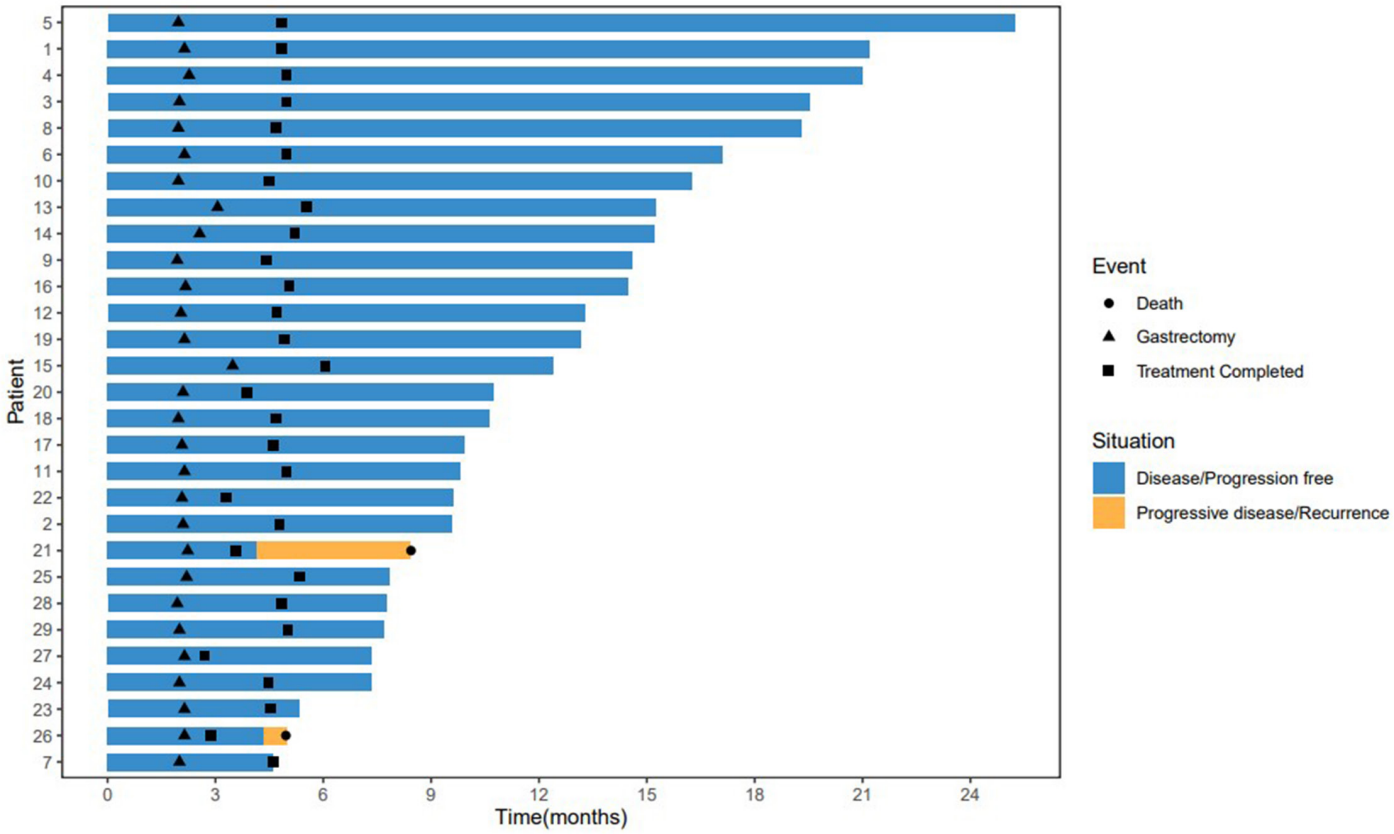
Swimmer plot of this trial (*n* = 29).

Until the data cut‐off date (December 3, 2022), with a median follow‐up time of 18.8 months (95% CI: 16.7–21.4 months), two patients died because of tumor recurrence, of whom one occurred malignant ascites 1 month after surgery, one experienced bone metastasis 2 months after surgery. The remaining patients are still under follow‐up. The median EFS and OS were not reached (Figure 2).

### 3.3 Adverse events

In total, 24 (82.8%) patients experienced treatment‐emergent adverse events (TEAEs). The most commonly reported TEAE of any grade was reactive cutaneous capillary endothelial proliferation (RCCEP) (10/29, 34.5%), followed by leucopenia (9/29, 31.0%), thrombocytopenia (9/29, 31.0%), nausea (5/29, 17.2%), and diarrhea (4/29, 13.8%). Immune‐related AEs were occurred in 13 patients, of which 10 (34.5%) patients developed RCCEP (grade I), two patients developed rash (2/29, 6.9%) (grade I), and one patient experienced pneumonia (grade II).

Three (10.3%) of 29 patients who underwent surgery experienced surgical complications such as chylous fistula, hypoalbuminemia, ascites, and infection of incision. No patients underwent reoperation. No patients died within 30 days, and one patient died within 90 days after surgery because of rapid disease progression (Table 5).

**TABLE 5:** Treatment‐emergent adverse events.

Events	Patients (*n* = 29)		
	Grade 1, *n* (%)	Grade 2, *n* (%)	Any grade, *n* (%)
Treatment‐emergent AEs, *n* (%)	19 (65.5)	5 (17.2)	24 (82.8)
RCCEP	10 (34.5)	0 (0)	10 (34.5)
Leucopenia	9 (31.0)	0 (0)	9 (31.0)
Thrombocytopenia	6 (20.7)	3 (10.3)	9 (31.0)
Nausea	5 (17.2)	0 (0)	5 (17.2)
Diarrhea	4 (13.8)	0 (0)	4 (13.8)
Hepatic dysfunction	1 (3.4)	1 (3.4)	2 (6.9)
Vomiting	2 (6.9)	0 (0)	2 (6.9)
Rash	2 (6.9)	0 (0)	2 (6.9)
Renal dysfunction	2 (6.9)	0 (0)	2 (6.9)
Pneumonia	0 (0)	1 (3.4)	1 (3.4)
Immune‐related AEs, *n* (%)	12 (41.4)	1 (3.4)	13 (44.8)
RCCEP	10 (34.5)	0 (0)	10 (34.5)
Rash	2 (6.9)	0 (0)	2 (6.9)
Pneumonia	0 (0)	1 (3.4)	1 (3.4)
Post‐operative complications	1 (3.4)	2 (6.9)	3 (10.3)
Chylous fistula	0 (0)	1 (3.4)	1 (3.4)
Hypoalbuminemia	0 (0)	1 (3.4)	1 (3.4)
Ascites	0 (0)	1 (3.4)	1 (3.4)
Infection of incision	1 (3.4)	0 (0)	1 (3.4)
30‐day mortality	0 (0)		
90‐day mortality	1 (3.4)		

Abbreviations: AE, adverse event; RCCEP, reactive cutaneous capillary endothelial proliferation.

## 4 DISCUSSION

In this prospective, single‐arm, phase II study, the primary endpoint of pCR was achieved in 3 (10.3%) patients, whereas 20 (69.0%) patients obtained MPR after receiving neoadjuvant therapy. Twenty‐eight (96.6%) patients obtained R0 resection, and 3 (10.3%) had tpCR. Meanwhile, no patients underwent reoperation, and no three or higher grade adverse events occurred by the data cut‐off date. To the best of our knowledge, this is the first phase II clinical trial investigating the efficacy and safety of neoadjuvant camrelizumab, oxaliplatin, and S‐1 followed by surgery in patients with resectable LAGC and GEJC.

Compared with surgery alone, neoadjuvant chemotherapy showed improved efficacy and has been recognized as a standard of care in patients with LAGC.^2^ Nowadays, the most commonly used neoadjuvant regimens for LAGC included combination of docetaxel, oxaliplatin, fluorouracil, and leucovorin (the FLOT regimen) and the SOX regimen.^16^ A phase II trial compared these two regimens in the Chinese population, and no significant differences were found between these two groups in clinical benefits.^17^ In Asian countries, the SOX regimen as perioperative treatment for resectable gastric cancers is more commonly used than the FLOT regimen, since it was considered more tolerant.^13^, ^18^, ^19^ In the RESOLVE study, a pCR rate of 5.6% was reported.^13^ Furthermore, in a recent phase III, randomized clinical trial, 3.4% patients in the SOX group achieved pCR, with 95.5% R0 resection rate.^20^ The pCR rate in our study was 10.3%, which was numerically higher than that in previous studies with SOX regimen.

Over the last decade, with the emergence of immunotherapy, accumulating evidence shows that adding ICIs to neoadjuvant chemotherapy could provide better clinical benefits than chemotherapy alone.^21^, ^22^ However, only a few phase II studies investigated neoadjuvant ICIs combined with chemotherapy in LAGC and GEJC patients, with pCR rates ranging from 19.4% to 25.0%, and MPR rates ranging from 44.4% to 63.3%.^23^, ^24^, ^25^, ^26^, ^27^, ^28^ Besides, in the randomized phase IIb DANTE study, the pCR rate was 25% in patients received neoadjuvant atezolizumab plus FLOT regimen, 24% in the neoadjuvant FLOT group, whereas the MPR rates between these two groups were 49% and 44%, respectively.^26^ Compared with these studies, our study presented a relatively lower pCR rate (10.3%), a higher MPR rate (69.0%), and improved safety profile. The difference of pCR rate may attribute to different patients' baseline characteristics such as the percentage of patients with signet‐ring cell carcinoma, ECOG status, and PD‐L1 expression level, as well as different chemotherapy regimens. In the present study, 24.1% (7/29) patients have signet‐ring cell carcinoma, much higher than in the phase II study with neoadjuvant sintilimab plus capecitabine and oxaliplatin (CapeOx regimen) (2.8%).^24^ The patients with ECOG performance status of 0 were 65.5% (19/29) in our study, lower than most of the above‐mentioned studies.^23^, ^24^, ^26^ In addition, only 17.2% (5/29) patients in our study had positive PD‐L1 status, whereas the percentage of patients with positive PD‐L1 status in the study with neoadjuvant sintilimab plus CapeOx regimen and the DANTE study were 58.3% and 56.3%, respectively.^24^, ^26^ Given most of these trials were single‐arm trials with relatively small sample size, the results should be compared and interpreted cautiously.

Regarding of predictive biomarkers, in our study, 5 (17.2%) patients had positive PD‐L1 expression at baseline and none of them achieved pCR after neoadjuvant treatment. Theoretically, positive PD‐L1 expression might be associated with better clinical effect when patients treated with ICIs.^29^ Several previous studies have shown that LAGC/GEJC patients with positive PD‐L1 have a higher chance of achieving pCR, but no definite conclusion can be drawn because of lack of randomized phase III studies, and potential bias may occur due to our small sample size.^24^, ^30^, ^31^

There are several limitations exist in our study: Firstly, our study is a single‐arm study without a control group; secondly, the patients in our study were recruited from a single‐center and the number was limited, which may cause potential bias; thirdly, the follow‐up of EFS and OS is still ongoing and the data are immature at present, further results are warranted to analyze survival benefits.

In conclusion, in the present study, neoadjuvant camrelizumab and oxaliplatin plus S‐1 show a modest clinical efficacy and acceptable safety profile, which needs to be verified in further studies in patients with LAGC or GEJC.

## AUTHOR CONTRIBUTIONS

**Wen‐Jin Zhong:** Conceptualization (equal); formal analysis (equal); investigation (equal). **Jian‐An Lin:** Data curation (equal); formal analysis (equal); software (equal). **Chu‐Ying Wu:** Formal analysis (equal); methodology (equal); validation (equal); writing – original draft (equal). **Jiantian Wang:** Formal analysis (equal); resources (equal); software (equal); writing – review and editing (equal). **Jun‐Xing Chen:** Investigation (equal); methodology (equal); software (equal); visualization (equal); writing – original draft (equal). **Huida Zheng:** Conceptualization (equal); data curation (equal); formal analysis (equal). **Kai Ye:** Conceptualization (equal); data curation (equal); investigation (equal); methodology (equal); project administration (equal); supervision (equal).

## FUNDING INFORMATION

None.

## CONFLICT OF INTEREST STATEMENT

The authors declare no conflict of interests.

## ETHICAL APPROVAL STATEMENT

The study protocol was approved by the institutional ethics committees of The Second Affiliated Hospital of Fujian Medical University. Written informed consents of all patients were obtained before study enrollment.

## CLINICAL TRIAL REGISTRATION NUMBER

The study was registered on clinicaltrials.gov (NCT05602935).

## Data Availability

Requests for de‐identified data may be directed to Kai Ye (zhongwj87@163.com).
